# The variability and burden of severe sleep apnea and the relationship with atrial fibrillation occurrence: analysis of pacemaker-detected sleep apnea

**DOI:** 10.1007/s11325-021-02385-1

**Published:** 2021-05-24

**Authors:** RuoHan Chen, KePing Chen, Yan Dai, Shu Zhang

**Affiliations:** Center of Arrhythmia, Fuwai Hospital, Chinese Academy of Medical Sciences, Peking Union Medical College, Beilishi Road 167#, Xicheng Qu, Beijing, 100037 China

**Keywords:** Respiratory disturbance index, Variability, Burden, Atrial fibrillation

## Abstract

**Study objectives:**

This was a pilot study to evaluate the long-term variability and burden of respiratory disturbance index (RDI) detected by pacemaker and to investigate the relationship between RDI and atrial fibrillation (AF) event in patients with pacemakers.

**Methods:**

This was a prospective study enrolling patients implanted with a pacemaker that could calculate the night-to-night RDI. The mean follow-up was 348 ± 34 days. The RDI variability was defined as the standard deviation of RDI (RDI-SD). RDI burden was referred to as the percentage of nights with RDI ≥ 26. The patient with RDI ≥ 26 in more than 75% nights was considered to have a high sleep apnea (SA) burden. An AF event was defined as a daily AF duration > 6 h.

**Results:**

Among 30 patients, the mean RDI of the whole follow-up period was 24.5 ± 8.6. Nine (30%) patients were diagnosed with high SA burden. Patients with high SA burden had a higher BMI (26.7 ± 4.8 vs 23.2 ± 3.9, *p* = 0.036), a higher prevalence of hypertension (86% vs 39%, *p* = 0.031), and a larger left ventricular diastolic diameter (49.2 mm vs 46.7 mm, *p* = 0.036). The RDI-SD in patients with a higher burden was significantly greater than that in the patients with less burden (10.7 ± 4.9 vs 5.7 ± 1.4, *p* = 0.036). Linear regression showed that participants with a higher RDI tended to have a higher SD (*R* = 0.661; *p* < 0.001). The mean RDI (OR = 1.118, 95%CI 1.008–1.244, *p* = 0.044) was associated with AF occurrence.

**Conclusion:**

Using a metric such as burden of severe SA may be more appropriate to demonstrate a patient’s true disease burden.

## Introduction

The apnea-hypopnea index (AHI) measured by polysomnography (PSG) is commonly utilized in diagnosing and classifying the severity of sleep apnea (SA). However, multiple studies have shown the intraindividual night-to-night variability of AHI in patients with SA. Bittencourt et al. demonstrated that the difference of AHI on consecutive nights would be greater than 10 events/h in 50% of patients [1]. The variability of AHI will not only compromise the precision of diagnosis and management but also affect the prognosis. Furthermore, there have been few studies regarding the assessment of AHI variability, and the results have been conflicting. Prasad et al. [2] reported that patients with mild SA (AHI 5–15/h) were associated with higher night-to-night variability. Aarab [3] showed that patients with higher AHI tended to have a higher variability. Among these studies, the discrepancy in results might be attributed to different monitoring technology (PSG vs portable monitor), test setting (sleep lab vs home), monitoring time (consecutive vs intermittent), and sample sizes.

Despite a high prevalence, SA has usually gone undiagnosed in patients with pacemakers [4, 5]. The pacemaker (PM) with transthoracic impedance sensor and novel algorithm is able to recognize ventilation pauses and reductions. The respiratory disturbance index (RDI) derived from PM has been closely associated with the diagnosis of SA determined by PSG [6, 7]. A PM may therefore continuously monitor indices of SA by providing night-to-night RDI, which makes it possible to evaluate the long-term variability of RDI. In addition, a PM may simultaneously record the data of RDI and atrial fibrillation (AF) events, affording an opportunity to evaluate the relationship between SA and AF.

The aims of this study were to evaluate the long-term variability of RDI in pacemaker patients and to investigate the relationship between RDI and AF event.

## Methods

### Pacemaker-based SA detection

**Fig. 1:**
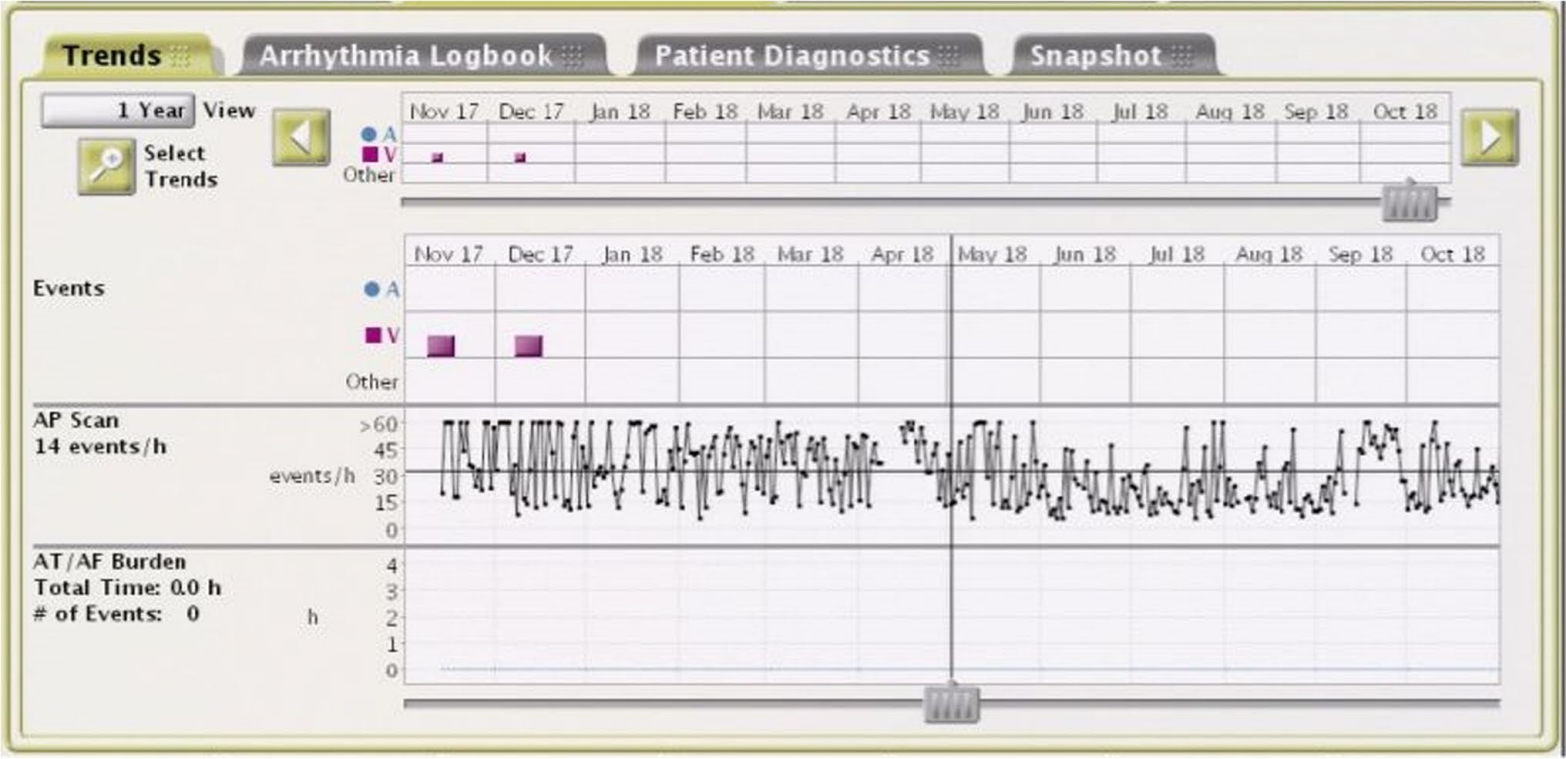
Example of respiratory disturbance index (RDI) display. The first row is the period of data storage. The second row is the atrial-ventricular high rate events. The third row is the trend of AP Scan. Y-axis represents the RDI (0 ~  > 60 events/h). The RDIs are plotted once a day to form the AP Scan trend. The RDI (14 events/h) for the day selected by the vertical axis is shown in the left column. The forth row is AT/AF burden. Y-axis represents the time (h) of AT/AF events

Recently, Boston Scientific/Vitalio pacemakers have been reported to have a transthoracic impedance sensor and AP Scan algorithm screen for SA in pacemaker patients [8]. Briefly, transthoracic impedance fluctuated regularly with respiratory movement. The difference of transthoracic impedance in a cycle may be utilized to estimate the variation in tidal volume. According to the AP Scan algorithm, respiratory disturbance event was defined as a decline of tidal volume of at least 26% from the baseline or a breathe suspended for at least 10 s or more [9]. An RDI was the average number of respiratory disturbance events the patients experienced per hour during a programmed sleep time. Sleep time was programmed individually according to patients’ sleep diaries. The RDIs were recorded and plotted once a day to form the AP Scan trend (Fig. 1).

### Patient population

This prospective single-center study was conducted at the Fuwai Hospital (Beijing, China). A series of consecutive patients undergoing implantation of a Vitalio J237 or J274 (Boston Scientific, St. Paul, MN, USA) from August 2016 to December 2017 were enrolled in the study, irrespective of whether or not the patients had symtpoms of SA or a SA diagnosis. All patients had an indication for pacemaker implantation according to current guidelines [10].

The exclusion criteria included clinical diseases affecting the transthoracic impedance sensor function or resulting in sleep alteration, such as chronic obstructive pulmonary disease, interstitial pulmonary diseases, heart failure, parasomnias, alcoholism, use of hypnotics, habits, or occupations leading to sleep deprivation or altered sleep–wake cycle. The respiratory signal amplitude could vary according to the posture or movement of patients. If the respiratory signal amplitude was unstable or signal-to-noise ratio was out of range, the RDI would then be discarded and the night would be considered invalid. Patients with > 10% invalid AP Scan data were excluded. Information including demography, underlying heart and pulmonary disease, pacing indication, atrial arrhythmia, and N-terminal pro brain natriuretic peptide (NT-proBNP) were obtained from medical record. All patients underwent standard two-dimensional and Doppler echocardiographic examination at rest. Measurements of the LV cavity dimension was derived from the parasternal long axis view of two-dimensional imaging. LVEDD was measured at end diastole (the onset of the QRS complex). LVEF was measured by modified Simpson rule from the apical four-chamber plane.

This study was approved by local ethics committees, and all enrolled patients were required to sign an informed consent.

### PSG measurements

After implantation. Patients underwent an attempt at overnight PSG. The overnight PSG was performed and interpreted by a sleep specialist who was blinded to the PM-RDI data. PSG examination included electroencephalogram (EEG) for sleep analysis, electromyogram for chest and abdominal respiratory movement, oral-nasal airflow, and arterial oxyhemoglobin saturation. A hypopnea event was defined by a decline of tidal volume at least 30% from baseline lasting 10 s or longer. An apnea event was defined by a suspension of oral-nasal airflow lasting 10 s or more. The PSG-AHI was the average number of apnea and hypopnea events per hour during sleep time. The severity of SA was graded by the AHI scores [11], that is, PSG-AHI ≥ 30: severe SA; 15 < or =  PSG-AHI < 30: moderate SA; 5 < or = PSG-AHI < 15: mild SA.

### RDI criteria, RDI variability, and RDI burden

RDI criteria has been described in our previous study [5]. That is, a 26 ≤ RDI < 41 identified patients as moderate SA, corresponding to 15 ≤ AHI < 30; an RDI ≥ 41 identified patients as severe SA, corresponding to AHI ≥ 30 with a sensitivity of 82.1%, a specificity of 88.6% [5]. The RDI data of the night during which time the patient underwent the PSG was deemed as the first night RDI. The mean RDI was equal to the average RDI during the follow-up.

During the follow-up, we found that the RDI, like other physiology parameters, such as heart rate and blood pressure, also fluctuated every day. We raised a concept as RDI variability, which was defined as standard deviation of RDI (RDI-SD). RDI burden was referred to as the percentage of nights with RDI ≥ 26.

### Atrial fibrillation event

The duration of AF each day was recorded in the pacemaker. Boriani’s study demonstrated that daily burden of 6 h was associated with 17% increase in the risk of stroke [12]. In Turakhia’s study, a burden of ≥ 5.5 h raised the short-term risk of stroke 4- to 5-fold [13]. In this study, an AF event was defined as the cumulative AF duration > 6 h in a day.

### Pacemaker follow-up and data extraction

The patients were routinely arranged follow-up at 1 month after implantation, every 6 months thereafter, and more frequently according to the clinical needs. The interrogations of the store data were performed during each follow-up. The store data included the RDI data and AF events. In this study, AF event was defined as the occurrence of AF lasting more than 6 h. All the data could be recorded in PM as long as 1 year. During the follow-up, the RDI data was restored in special memory card and sent to technical department of Boston Scientific company to be extracted automatically by special software.

### Statistics

The patients were divided into two groups according to the percentage of night with the mean RDI ≥ 26. Higher burden of severe SA indicated the patients with more than 75% nights RDI ≥ 26. The less burden of severe SA suggested the patients with either mean RDI ≤ 26 or mean RDI ≥ 26 but burden < 75%. Categorical variables were presented as the number and percentage and were compared via chi-square analysis or Fisher’s exact test. Continuous variables were presented as mean ± SD if conform to normal distribution and presented as median (quartile) if not. And independent *t* test was applied to compare continuous variables. Standard deviation (SD) was used to appraise the variability of RDI. Linear regression was calculated between the individual’s mean RDI and SD. The univariate analysis and multivariable logistic regression logistic regression was used to analyze the relationship between RDI and AF occurrence with the Forward method. The variables included AHI, mean RDI, and first night RDI. Age and BMI were also associated with AF occurrence; these two variables were included in the logistic regression analysis. The Kolmogorov–Smirnov test was applied for testing the fit of variables to a normal distribution, including mean RDI, AHI, and first night RDI. All statistical analyses were performed with IBM SPSS 19.0 package program. A *p* value of < 0.05 was considered significant difference.

## Results

### Patient population

A total of 35 patients underwent Vitalio pacemaker implantation, among them 5 patients (14.3%) were excluded due to least 10% invalid nights, with the rest 30 patients being included. The mean age was 65.1 ± 9.8 years, and 15 (50%) were men. The pacing indication was sick sinus syndrome. No patients had documental SA diagnosis or underwent PSG exam before PM implantation. Fourteen (44.8%) patients had a history of paroxysmal AF. All the patients underwent a dual-chamber pacemaker implantation. Twenty-three (76.6%) patients had an AHI ≥ 5 (8, mild; 9, moderate; 6, severe), and the average AHI was 16.1 ± 12.7. The patients’ characteristics are presented in Table 1.

**Table 1:** Patients’ characteristics

	Total *N* = 30	Higher burden *N* = 9	Less burden *N* = 21	*p* value
Age (yr)	65.1 ± 9.8	66.4 ± 12.6	64.7 ± 9.1	0.692
Male (%)	15 (50%)	5 (71.4%)	10 (43.5%)	0.195
BMI	23.8 ± 3.9	26.7 ± 4.8	23.2 ± 3.4	0.036
Smoke	9 (30.0%)	1 (14.3%)	8 (34.8%)	0.300
Hypertension (%)	15 (50.0%)	7 (85.7%)	9 (39.1%)	0.031
Coronary atrial disease (%)	8 (26.7%)	1 (14.3%)	7 (30.4%)	0.398
Diabetes (%)	4 (13.3%)	1 (14.3%)	3 (13.0%)	0.933
Heart failure	8 (26.7%)	2 (28.6%)	6 (26.1%)	0.896
Paroxysmal AF history (%)	14 (46.7%)	4 (57.1%)	10 (43.5%)	0.526
AF during follow-up	12 (40.0%)	5 (71.4%)	7 (30.4%)	0.050
LVEDD	46.7 ± 3.3	49.2 ± 3.2	46.0 ± 3.0	0.036
Ejection friction	63.8 ± 4.0	64.3 ± 3.8	63.7 ± 4.2	0.742
AHI	16.1 ± 12.7	25.7 ± 17.8	13.3 ± 9.7	0.031
NT-proBNP	163.5 ± 214.6	167.7 ± 175.8	162 ± 228.6	0.938
RDI of first night	25.4 ± 13.2	38.0 ± 9.8	21.8 ± 9.7	0.006
Mean RDI	24.5 ± 8.6	35.3 ± 7.3	21.2 ± 5.9	0.001
RDI-SD	6.9 ± 3.3	10.7 ± 4.9	5.7 ± 1.4	0.009

*BMI* body mass index, *AF* atrial fibrillation, *LVEDD* left ventricular end diastolic diameter, *AHI* apnea/hypopnea index, *RDI* respiratory disturbance index, *RDI-SD* standard deviation of respiratory disturbance index

### RDI values, variability, and burden

**Fig. 2:**
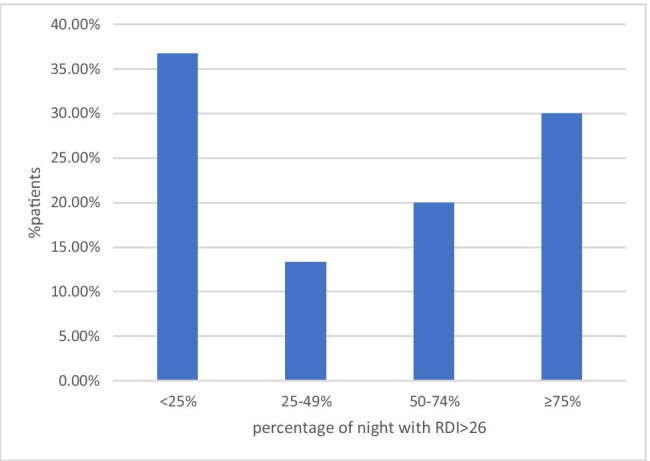
SA burden. Distribution of patients according to the percentage of their nights with RDI > 26

The patients were followed for a mean period of 356 ± 27 days (range 278–365 days). From 10,442 nights, 10,003 (95.8%) nights’ RDI data were extracted for further analysis. Only 3 patients (10%) never had a single RDI greater than 26, 27 patients (90%) had at least 1 night with RDI ≥ 26, and 17 patients (56.6%) had at least 1 night with RDI ≥ 41. Fifteen patients (50%) presented with more than 50% of nights with RDI ≥ 26 (Fig. 2). The average of the first night RDI was 27.8 ± 13.3. The overall mean RDI of the whole follow-up period was 24.5 ± 8.6. As regards the SA diagnosis, there was no significant difference between the first night RDI and the mean RDI (*p* = 0.192).

Nine (30%) patients were diagnosed as the high SA burden, as the RDI ≥ 26 in more than 75% nights. Compared with the less burden patients, the high burden patients were found with a higher BMI (26.7 ± 4.8 vs 23.2 ± 3.9, *p* = 0.036) and a higher prevalence of hypertension (85.7% vs 39.1%, *p* = 0.031). The left ventricular diastolic diameter was larger in high burden group than that in less burden group (46.7 mm vs 49.2 mm, *p* = 0.036). Despite the fact that AF occurrence during the follow-up was more common in high burden group (71.4% vs 30.4%, *p* = 0.05), there was no significant difference in history of paroxysmal AF between the two groups (57.1% vs 43.5%, *p* = 0.526). The mean RDIs in higher and less burden groups are 35.3 ± 7.3 and 21.2 ± 5.9, respectively (*p* = 0.001). The RDI-SD among all patients is 6.9 ± 3.3. The RDI-SD in higher burden patients is significantly greater than that in the less burden patients (10.7 ± 4.9 vs 5.7 ± 1.4, *p* = 0.036) (Table 1).

**Fig. 3:**
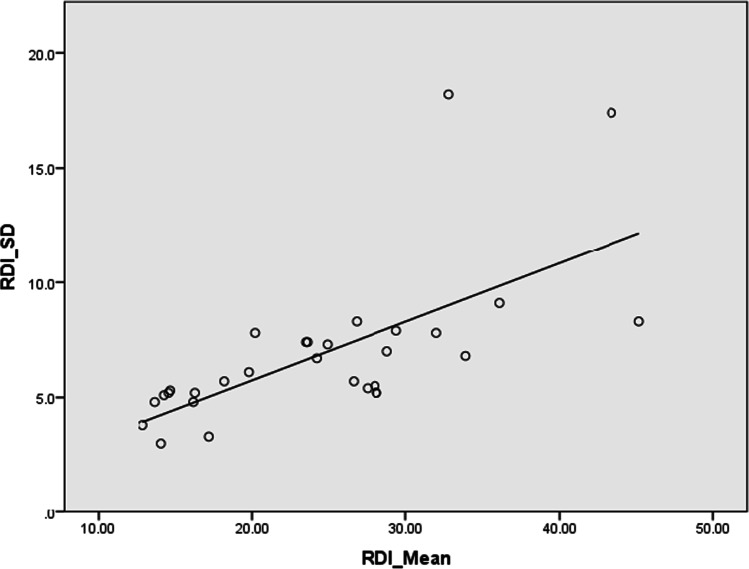
Linear regression between the mean and the SD of RDI, which with a tendency of higher RDI and higher RDI-SD

Linear regression demonstrated a significant linear relationship between the mean RDI and the RDI-SD (*R* = 0.661; *p* < 0.001), with a tendency of higher RDI and higher RDI-SD (Fig. 3).

### Relationship between RDI and AF onset

During the follow-up, 12 patients (40%) experienced at least an AF episode lasting more than 6 h. K-S test confirms that the mean of RDI, AHI, and first night RDI comply with the normal distribution. After adjusting for age and BMI in logistic regression model, the mean of RDI (OR = 1.118, 95%CI 1.008–1.244, *p* = 0.044) had an increased risk of AF occurrence, while the first night RDI (OR = 1.001, 95%CI 0.942–1.063, *p* = 0.982) or the AHI (OR = 1.015, 95%CI 0.954–1.080, *p* = 0.637) could not predict the onset of AF.

## Discussion

In this study, we demonstrate the high RDI burden and variability in PM patients. Compared with that in less SA burden patients, the long-term variability of RDI is greater in high SA burden patients. The mean RDI is associated with AF occurrence during the follow-up.

Many studies have confirmed the high prevalence of SA in pacemaker patients. By PSG examination, the percentage of SA in pacemaker patients could be 60 ~ 78% [4, 6]. In this study, 76.6% of patients had SA with AHI ≥ 5/h, but none of them had been diagnosed as SA or undergone PSG exam. Previous studies showed that the RDI calculated by transthoracic impedance sensor and novel algorithm could be used to identify SA in PM patients, with a sensitivity and specificity of both 90%. Our study showed the RDI could be measured during at least 90% of all night in 85.7% of patients during 1 year period. In Moubarak study, the function of pacemakers recording the RDI was assessed in 6 months. According to the results, RDI could be measured with high quality in 98% of night in 89% of patients [14]. The reliability of RDI measurement by PM was important, serving as a prerequisite for value of RDI in potential SA patients management and clinical studies.

In this study, 15 patients (50%) were diagnosed as moderate or severe SA by PSG, while being continuously monitored by PM, about 90% patients had an RDI ≥ 26 at least one night during 1 year period. Six patients (20%) were diagnosed as severe SA, but more than 56% of patients had at least one night with RDI ≥ 41. These phenomena could be explained by the variability of RDI. By repeating PSG exams, the variability of AHI was demonstrated in previous studies. In Aarab et al. study, the detectable difference for AHI was 12.8/h, suggesting that presence or absence of SA and its severity could not be reliably detected by a single measurement, unless AHI was in an extreme range (< 0.2/h or > 9.8/h). Other than some extrinsic factors, such as medications, alcohol consumption, the body positions, and sleep architecture, the intrinsic variability in SA also results in AHI variability. Therefore, the variability in AHI could not be eliminated by repetition of test, but its estimation would be more accurate. However, repetitive PSG is not clinically practical for each patient. In addition to the PSG being expensive and time-consuming, the availability of sleep labs hampered its clinical application. Some convenient, portable, and continuous device measurement would be more useful in SA patient assessment.

In this study, the variability of RDI mirrors the variability in AHI determined by PSG. The RDI-SD in this study was 3.3. This figure was much smaller than AHI-SD in previous study, which was about 24 ~ 36 [15]. Considering that the figure of RDI was larger than that of AHI in SA diagnosis (26 vs 15, respectively), the RDI-SD was less obvious in this study than the AHI-SD in previous study. This could be partly explained by the difference of the frequency of exam. In this study, the RDI-SD was calculated from 1-year data. In previous AHI study, the AHI-SDs were calculated from a maximum of 4 PSG exams. Our study also demonstrated the significant linear relationship between the mean RDI and the RDI-SD. The patients with a lower mean RDI were more likely to smaller variability in comparison with those with a higher mean RDI. Similar trend in severe SA were observed in the other study [3].

The variability of SA confirmed that the presence of SA and its severity could not be reliably detected by a one-night measurement. The mean RDI and the burden of RDI might be more appropriate to demonstrate the patient’s true disease burden. As the RDI could be calculated and recorded every day by PM, it provided us with a convenient way to reveal the presence and severity of SA in pacing-indicated patients. And further studies are needed to go in depth with the data analysis to have a better understanding of RDI variability, such as the periodicity of the RDI variability according of seasons etc.

In our study, the patients with RDI ≥ 26 in more than 75% nights were considered as higher burden patients. The higher SA burden patients are more in weight and with higher prevalence of hypertension. These two characteristics were also associated with higher incidence of SA in the general population. AF also occurred more frequently in higher burden group even though there was no significant difference in paroxysmal AF history between the two groups. OSA facilitated the onset of AF via many mechanisms, including hypoxia/hypercapnia, atrial enlargement, alternations in autonomic tense, and inflammation [16]. Continuous positive airway pressure therapy in SA patients could lower the recurrence of AF. In Gami et al. study, SA detected by PSG was more prevalent in AF patients [17], followed by the same authors conducting a retrospective study, with the results showing that SA was an independent risk factor for AF occurrence in patients without AF history over a 4-year follow-up [18]. Mazza’s study [19] demonstrated that severe SA detected by PM was associated with higher risk of AF in patients with or without AF history, and the severity of SA could predict AF occurrence within the next 3 months. Our study also demonstrated that the mean RDI is associated with AF occurrence, while AHI or the first night RDI could not predict the onset of AF.

### Study limitations

Limitations of this study included the small number of patients and solo pacing indication. As we wanted to appraise the long-term variability of RDI, it would be more interesting to consider longitudinal observations instead of using an aggregated measure such as mean and SD. Besides a mixed model instead of a linear regression could improve the power of the study. In order to evaluate the relationship between the AF event and RDI, we excluded the patients with persistent AF, and all the patients underwent dual-chamber pacemaker implantation. This resulted in atrioventricular block patients being excluded from this study and might come with some systemic error. In addition, in this study, patients were considered to have AF event if the device detected that AF burden was greater than 6 h, which may contribute to the underestimation of AF events.

## Conclusion

SA is a common syndrome but usually remains undiagnosed in pacemaker recipients. The RDI by PM may be used to appraise the intrinsic variability of SA, which has a linear relationship with the mean RDI. The burden of high RDI would be more reliable than one-night measurement in terms of the presence and severity of SA. The mean RDI was associated with AF events.
